# Improved antifouling properties and selective biofunctionalization of stainless steel by employing heterobifunctional silane-polyethylene glycol overlayers and avidin-biotin technology

**DOI:** 10.1038/srep29324

**Published:** 2016-07-06

**Authors:** Ville Hynninen, Leena Vuori, Markku Hannula, Kosti Tapio, Kimmo Lahtonen, Tommi Isoniemi, Elina Lehtonen, Mika Hirsimäki, J. Jussi Toppari, Mika Valden, Vesa P. Hytönen

**Affiliations:** 1 BioMediTech, University of Tampere, Biokatu 6, FI-33520 Tampere, Finland; 2 Surface Science Laboratory, Optoelectronics Research Centre, Tampere University of Technology, P.O. Box 692, FI-33101 Tampere, Finland; 3 University of Jyvaskyla, Department of Physics, NanoScience Center, P.O. Box 35, FI-40014 University of Jyväskylä, Finland; 4 Fimlab Laboratories, Biokatu 4, FI-33520 Tampere, Finland

## Abstract

A straightforward solution-based method to modify the biofunctionality of stainless steel (SS) using heterobifunctional silane-polyethylene glycol (silane-PEG) overlayers is reported. Reduced nonspecific biofouling of both proteins and bacteria onto SS and further selective biofunctionalization of the modified surface were achieved. According to photoelectron spectroscopy analyses, the silane-PEGs formed less than 10 Å thick overlayers with close to 90% surface coverage and reproducible chemical compositions. Consequently, the surfaces also became more hydrophilic, and the observed non-specific biofouling of proteins was reduced by approximately 70%. In addition, the attachment of *E. coli* was reduced by more than 65%. Moreover, the potential of the overlayer to be further modified was demonstrated by successfully coupling biotinylated alkaline phosphatase (bAP) to a silane-PEG-biotin overlayer via avidin-biotin bridges. The activity of the immobilized enzyme was shown to be well preserved without compromising the achieved antifouling properties. Overall, the simple solution-based approach enables the tailoring of SS to enhance its activity for biomedical and biotechnological applications.

In biotechnology and food and pharmaceutical industries the ability to specifically adjust and modify a material’s interactions with its surroundings is extremely desirable^1^,^2^,^3^. Unfortunately, materials with both beneficial bulk properties and appropriate surface functionalities are seldom readily available. Therefore, surface modification of materials that demonstrate otherwise potential properties, such as stainless steel (SS), is often preferable^2^,^4^. For example, through covalent coupling reactions and polymer grafting, otherwise biologically challenging materials can be adjusted to comply with various circumstances^5^,^6^.

SS is a commonly used metal in industrial and medical settings, where it becomes exposed to organic and biological agents that may result, for example, in biofouling and microbial-induced corrosion^7^. For instance, orthopedic implants and cardiovascular stents are constantly in contact with bodily fluids and interact with living cells^1^,^8^,^9^. Among others, SS has distinguished thermal and mechanical properties, great workability, availability, and inherent corrosion resistance, which results from its native, self-healing oxide layer^3^. The oxide layer also contributes to the biocompatibility of SS^10^. However, for optimal biological performance biofunctionality is also required. For instance, enzymes, such as lysozyme and alkaline phosphatase have been used to introduce antibacterial properties or other specific biological functions to surfaces^11^,^12^.

Silanes are well known and widely used surface-functionalization agents that are able to bind to the hydroxide groups of the SS oxide layer, and in suitable conditions strictly organized monolayers can be formed^12^,^13^. For example, Slaney *et al*.^13^ have used atomic layer deposition (ALD) to precoat SS surfaces to facilitate the subsequent addition of bimolecular silane layers. In addition, Si-O-Si siloxane bonds can be formed between adjacent silane molecules, which adds to the tenacity of the overlayer^12^,^14^,^15^. Various types of functionalized silanes, such as the heterobifunctional silane-polyethylene glycol (silane-PEG) used in this study, are also readily commercially available and in general they are relatively inexpensive and considered environmentally safe.

Here, we report a simple and straightforward solution based method to deposit an extensive silane-PEG overlayer on an SS substrate to alter its biofunctionality. The approach is based on the protocol previously reported in Vuori *et al*.^12^. However, instead of implementing a step-by-step buildup of the silane overlayer, ready-made heterobifunctional silane-PEGs with either carboxylic acid (silane-PEG-COOH, SPC) or biotin (silane-PEG-biotin, SPB) as the other end group were used. The concept of the study is depicted in Fig. 1. In addition to surface biofunctionality, special care was placed on the extensive characterization of the surface with high resolution photoelectron spectroscopy methods complemented with imaging techniques.

Initially, SS was electrochemically (EC) hydroxylated to ensure the presence of an extensive oxide layer. This surface pretreatment is essential for the proper silanization of SS and imposes additional challenges and reaction steps when compared to, for example, the modification of more reactive and responsive silica^16^,^17^ and glass surfaces^18^,^19^. Insufficient surface pretreatment might lead to the undesired aggregation of the silane-PEGs and, consequently, result in an uneven silane overlayer. Since we have shown in Vuori *et al*.^12^ that EC treatment generates a high-quality, reproducible and largely contaminant-free passivated surface and we have solid experience with the technique, EC treatment was selected as the surface pretreatment method to be used here. Silanization of the EC-treated SS was then performed in one step by simple immersion technique, which makes the procedure extremely user friendly. Due to the silane groups, the silane-PEGs bind SS first via hydrogen bonds, which may then be converted into more rigid covalent bonds^20^. SPC molecules were used to improve SS antifouling properties, whereas SPB overlayers enabled further specific immobilization of avidin (neutral chimeric avidin, nChiAvd^21^) onto the surface. Characteristically, avidin binds very tightly to the biotin molecules available on the surface and, also, allows for further addition of other biotinylated molecules^22^. Consequently, avidin-biotin bridges were used to functionalize the surface with biotinylated alkaline phosphatase. All in all, the extent of modifications was assessed with various surface sensitive techniques, the antifouling properties with protein adsorption and bacterial adhesion tests, and the achieved biofunctionality with spectroscopic enzyme activity assay^12^.

## Methods

### Materials

Laser-cut electrochemically polished 12.5 mm × 28 mm × 0.7 mm EN 1.4404 (AISI 316L) SS chips produced by Outokumpu Stainless Oy (Tornio, Finland) were ordered from Kaarinan Trimet Oy (http://www.kaarinantrimet.fi/). Silane-PEG-COOH (SPC, MW 2000 Da) and silane-PEG-biotin (SPB, MW 2000 Da) were acquired from Nanocs Inc. (New York, NY, USA). Sulfuric acid, toluene and phosphatase substrate (pNPP, Product No. S0942) were bought from Sigma-Aldrich (St. Louis, MO, USA), and glacial acetic acid and acridine orange zinc chloride double salt (Product No. 115931) from Merck (Darmstadt, Germany). Wildtype chicken avidin (Belovo, Belgium, MW = 16 kDa/monomer) and fibronectin (gelatin-affinity purified from human serum, MW = 220 kDa/monomer) had been previously labeled with Alexa Fluor® 488 NHS ester (Cat. No. A-20000, Thermo Fisher Scientific, Inc. Waltham, MA, USA) by Dr. J. Pärssinen according to manufacturer’s instructions. Chemically competent *E. coli* Top10 cells were acquired from Thermo Fisher scientific, Inc. For biofunctionalization, neutral chimeric avidin (nChiAvd^21^) and biotinylated alkaline phosphatase (Cat. No. B-2005, Vector Laboratories (Burlingame, CA, USA) were used. Vectashield Hardset Antifade Mounting Medium (Cat. No. H-1400, Vector Laboratories) was used for microscope sample preparation.

### Hydroxylation

An SS chip was washed by sonication in both ethanol and deionized (DI) water for 10 min each at room temperature (RT). EC treatment was performed using an Autolab PGSTAT12 potentiostat/galvanostat (Methrom Autolab, The Netherlands) with a three-electrode electrochemical cell, the SS sample as a working electrode, an Ag/AgCl reference electrode and an SS (316L) counter electrode. N_2_ degassed 0.1 M sulfuric acid was used as electrolyte. Mild degassing was continued throughout the EC treatment. The SS sample was reduced for 10 min with a constant cathodic current of 5 mA/cm^2^ followed by passivation for 10 min with a constant potential of E_p_ = 0.2 V against the Ag/AgCl reference electrode. The sample was washed by rinsing with DI water and dried under an N_2_ stream.

### Silanization

The silane terminus of the silane molecules is triethoxy silane for both SPC and SPB. Silane-PEG (SPC or SPB) was dissolved in toluene at the desired concentration (3 or 5 mg/ml). An SS chip was immersed in the solution for approximately 43 h (two nights) on a rocking shaker at RT. The solution was removed and the chip annealed under atmospheric conditions at 100 °C for 10 min. The chip was rinsed with a stream of toluene by using a pipette, allowed to air-dry, and then washed by immersion in an excess of water three times for at least 30 s with intense shaking (230–250 rpm). Finally, the sample was air dried under laminar flow.

### Contact angle measurements

A custom-made imaging system with Pisara image-analyzing software (FotoComp Oy, Jyväskylä, Finland) was used for contact angle measurements. Drops of 4 μl of DI water, 7–12 drops for each SS chip, were used. The samples were imaged immediately after pipetting the drops to the chips. Contact angles for SS-SPC, SS-SPB, and unmodified SS were determined.

### Photoelectron spectroscopy (PES)

PES measurements were conducted in two different analysis systems: one featured a non-monochromatized Al Kα radiation (photon energy hν = 1486.6 eV) source (XPS)^23^ and the other utilized synchrotron (SR-PES) at the D1011-beamline D1011 of MAX II storage ring in the MAX IV Laboratory (Lund, Sweden). The detection area for the XPS analyses was approximately 0.28 mm^2^. The SR-PE spectra were collected by a SCIENTA SES-200 electron energy analyzer in FAT mode with 200 eV pass energy at a normal emission angle. The total energy resolution was ~100 meV and the detection area for the SR-PES analyses was approximately 0.04 mm^2^.

The surface chemical states were identified by analyzing high-resolution PE spectra. Upon subtracting the linear backgrounds, the spectral components were fit with a combination of Gaussian and Lorentzian line shapes (GL30) using Casa XPS software version 2.3.16^24^. The binding energies were calibrated to 285.0 eV for aliphatic carbons.

The surface morphologies of the samples were determined by inelastic electron energy background (IEEB) analysis using QUASES-Tougaard software package^25^,^26^,^27^, where the attenuation of Fe 2p (hν = 1486.6 eV) intensity of the silanized samples was compared to that of the SS-EC 12,14,15. The error of the method was estimated to be 15%^28^. The IEEB analysis is described in further detail in Supplementary Information.

### Atomic force microscopy (AFM)

AFM measurements were performed with a Dimension 3100 (Bruker Corporation, Billerica, MA, USA) together with Nanoscope Analysis software (Bruker) and Aspire CT300 Conical tapping mode AFM probes (Part No. CT300R-25, Nanoscience Instruments, Phoenix, AZ, USA). Phase and height images were taken in air using tapping mode.

### Protein adsorption

SS-SPC chips were affixed onto a microscope slide with double-sided adhesive tape and flexiPERM micro12 chambers (Sarstedt, Nürnbrecht, Germany) were secured thereupon to form well plates. Fluorescently labeled avidin and fibronectin were added to the wells at two different concentrations (3 or 30 μg/ml in PBS) for 1 or 3 h in the dark at RT. The labeling ratios of the proteins were 0.47 and 8.1 molecules of dye per protein subunit for avidin and fibronectin, respectively. Unmodified SS was used as reference. The samples were washed three times with an excess of PBS, flexiPERM chambers removed, and then air-dried. Glass coverslips were mounted onto the samples, and they were imaged with an LSM-780 confocal microscope equipped with Zen Black software (Zeiss, Germany) using 10× objective (Plan-Apochromat 10×/0.45 M27). Five images per flexiPERM well area were taken from random locations. The mean intensities of the images were recorded as obtained from the Zen Black software. Fixed exposure and gain settings, initially adjusted to prevent overexposure, were used to allow for comparison of the intensity values. The background intensity measured for an SS-SPC sample immersed in clean PBS was subtracted from the measured intensities of all the samples.

### Adhesion of *E. coli*

*E. coli* Top10 cells were precultured in LB medium overnight on a platform shaker (150 rpm) at 37 °C. The optical density of the culture solution at 600 nm (OD_600_) was measured with a BioPhotometer Plus instrument (Eppendorf, Hamburg, Germany), and it was adjusted to OD_600_ = 2.0 by diluting the samples with LB medium. SS-SPC was immersed in the bacterial suspension and incubated for 1 or 6 h on a rocking shaker at RT. Unmodified SS was used as reference. The sample placed in 6-well plate was washed three times for 30 s by immersion in 1.5 ml of PBS under stirring (230–250 rpm) to remove loosely attached bacteria, and was then air-dried. The bacteria were fixed and stained by immersing the sample in 3 mg/ml acridine orange in 2% glacial acetic acid for 2 min in the dark at RT. The sample was attached to a microscope slide with double-sided adhesive tape and a coverslip was mounted onto it. Imaging was performed with an LSM-780 confocal microscope (Zeiss, Germany) with Zen Black software using a 63× oil immersion objective (Plan-Apochromat 63×/1.40 Oil DIC M27). Mosaic images (10 × 10) were taken from random locations on the samples, and the amount of bacterial cells per image was calculated using ImageJ^29^.

The adhesion of *E. coli* on SS was also evaluated with scanning electron microscopy (SEM). Here, 3 mg/ml SS-SPC samples and an unmodified SS reference were used. *E. coli* had been fixed on the surfaces with 4% paraformaldehyde. The samples were coated with a 4 nm layer of a gold/palladium mixture (3 + 1) using an ultra-high vacuum electron beam evaporator (Instrumentti Mattila, Mynämäki, Finland). For the imaging, a Raith eLiNE 50 system (Raith Inc., Dortmund, Germany) was used at an acceleration voltage of 10 kV.

For comparison, bacterial adhesion tests were also conducted on glass and BSA-coated glass. Coverslips (18 × 18 mm; Zeiss) were used as substrates. Before the experiments, the coverslips were washed by successively immersing them in 0.1 M NaOH, H_2_O, 70% ethanol and H_2_O; then, the coverslips were air-dried. For the BSA coating, a 100 μg/ml BSA in H_2_O was prepared. The solution was added onto the coverslips for 1 h at RT. The coverslips were then washed three times with an excess of PBS and air-dried. The bacterial adhesion tests were conducted as described above.

### Biofunctionalization with avidin

3 μg/ml of neutral chimeric avidin^21^ in PBS was added onto SS-SPB for 1 h at RT. The samples were washed six times with an excess of TBS-tween20 (Tris-buffered saline with 0.05% Tween20) under vigorous shaking. The attachment of avidin was assessed with AFM. To biofunctionalize the SS-SPC-nChiAvd, biotinylated alkaline phosphatase was diluted 1:5000 in 100 mM Tris-HCl + 150 mM NaCl buffer (pH 7.5). This solution was added onto the SS-SPC-nChiAvd for 1 h at RT. The samples were washed six times with an excess of TBS-tween20 with vigorous shaking to remove unbound enzymes. Subsequently, 50 μl drops of 1 mg/ml of pNPP phosphatase substrate in 1 M diethanolamine (DEA) buffer containing 0.5 mM MgCl_2_ (pH 9.8) were applied onto the surfaces and the samples were protected from light. 2 μl samples were then obtained from the drops at time points of 10, 20, 30, 40, 50 and 60 min after the addition of pNPP to measure the respective absorbances at 405 nm using a NanoDrop 2000 Spectrometer (Thermo Scientific, Wilmington, DE, USA). Neutral chimeric avidin blocked with 2 μM free biotin was used to prepare the reference samples.

## Results and Discussion

### Topography and chemical properties of silane-peg-modified SS

**Figure 2:**
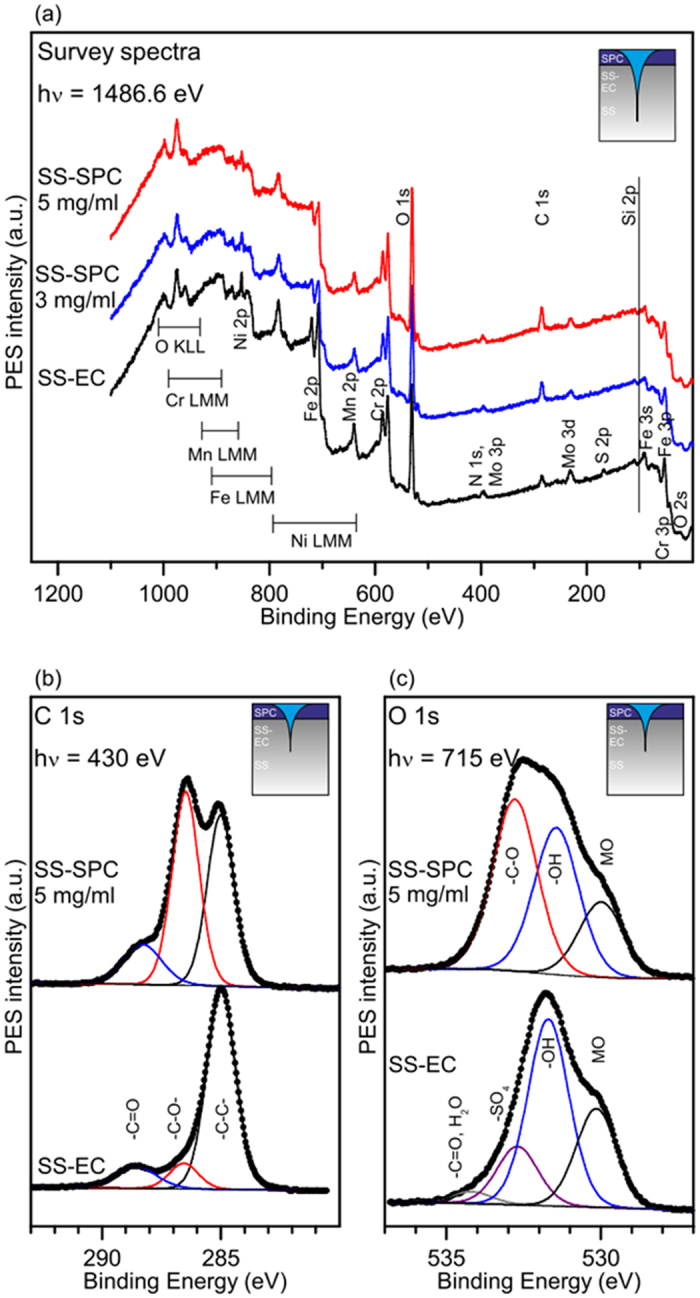
Photoelectron analyses of the silane-PEG-modified SS. (**a**) XPS survey spectra of the SS-EC and SPC-SS samples. (**b**) C 1s SR-PE spectra (hν = 430 eV) (**c**) and O 1s SR-PE spectra (hν = 715 eV) of the SS-SPC and SS-EC samples. The spectra shown in (**b,c**) are normalized. The sampling depths are schematically illustrated in the inset.

Photoelectron spectroscopy results are shown in Fig. 2. Figure 2(a) depicts the survey spectra of the SS-EC (SS after EC treatment) and SS-SPC samples with 3 and 5 mg/ml of SPC examined with conventional XPS (hν = 1486.6 eV). The sampling depth for each elemental PES transition depended on the kinetic energy of the photoelectrons and the material parameters. The sampling depth (i.e., 95% of the PES intensity originates from this depth) in Fig. 2(a) varied between ~6 and 10 nm. Hence, the survey spectrum consisted of photoelectrons from the SPC overlayer, the oxide layer of the SS surface and, to a small degree, the SS bulk phase. In the SS spectrum, elements of 316L stainless steel were detected (i.e., Fe, Cr, Ni, Mn, Mo, S, N and C). On the SS-SPC, an increased amount of carbon was observed. From the survey spectra, it was evident that the SPC overlayer was rather thin on the SS substrate because the signals from the metallic elements of the SS were not attenuated to a great degree on the silanized samples. High-resolution conventional XP spectra (hν = 1486.6 eV) showed that Si was present on the SS-SPC samples (See Supplementary Tables 1 and 2).

With conventional XPS, the experiments were limited to one or two photon energies, and therefore only a few different sampling depths could be determined for each element if they were operated at a constant emission angle. SR-PES provides an excellent method for probing into the surface chemistry of thin organic coatings on solid substrates because the energy of the photon flux that is used for the emission of photoelectrons can be selected from a continuous range of photon energies. Therefore, with SR-PES, it was possible to select the sampling depth of emitted electrons for each element. Also, the SR-PES enables the better recognition of the chemical states due to higher energy resolution. In Fig. 2(b,c), the SR-PES transitions of C 1s and O 1s are presented, respectively. The photon energies were chosen to yield high surface sensitivities. For both C 1s and O 1s, the sampling depths were approximately 2 nm. Figure 2(b) shows the C 1s SR-PE spectra (hν = 430 eV) of the SS and SS-SPC (5 mg/ml) samples. The C 1s spectrum for SS (bottom) was typical for stainless steel that has been exposed to atmospheric conditions^15^. The peaks at 285.0, 286.5 and 288.3 eV could be assigned to the C-O and C=O bonds, respectively. The presence of the SPC molecules on the SS-EC surface (top) was evident from the significant increase in the C-O intensity after silanization. Figure 2(c) demonstrates that the O 1s SR-PE spectrum (hν = 715 eV) of SS-EC (bottom) consisted of metal oxides at 530.1 eV, hydroxides at 531.6 eV, sulfates at 532.6 eV and organic impurities or H_2_O at 534.1 eV^15^. The large amount of hydroxides and the sulfate residues originated from the EC treatment^14^,^15^. The O 1s spectrum of SS-SPC exhibited three peaks at 530.0 eV (metal oxides), 531.4 eV (hydroxides) and 532.8 eV. The peak at 532.8 eV could be assigned to the C-O bonds in PEG^30^. Metal oxides and hydroxides at the SS surface were attenuated upon the adsorption of SPC. Sulfate residues and possible Si-O-Si bonds were also observed in the same binding energy range as the C-O bonds in PEG, and hence could not be resolved. Thus, the extensive covalent siloxane bonding of the overlayer could not be confirmed by SR-PES. However, no considerable deterioration of the surface coatings were detected during the experiments, which suggest tight attachment of the silane-PEGs. Moreover, there were no significant differences in the relative elemental surface concentrations and chemical states between samples with 3 mg/ml and 5 mg/ml or between heated and unheated samples (see Supplementary Table S2).

Nonetheless, IEEB analyses were used to determine surface morphologies. The best fit was obtained using a surface morphology with a thin silane overlayer in addition to low coverage with highly clustered islands with thicknesses >200 Å (see Supplementary Figure S1). This was in accordance to our earlier observations of thinner silane monolayers (more specifically aminopropyl trimethoxysilane and mercaptopropyl trimethoxysilane) on stainless steel^12^,^15^. A possible reason for the formation of clustered islands is the structural irregularities of the SS substrate^15^. The thickness of the 3 mg/ml SS-SPC sample overlayer was 4.6 Å with 86.9% coverage and that of the 5 mg/ml SS-SPC sample overlayer was 7.7 Å with 83.2% coverage (see Supplementary Table S3). These IEEB analyses indicated that the entire surface of the SPC-SS surface was covered with SPC molecules.

The thicknesses of the deposited silane-PEG layers were close to the thickness of APS coatings reported in Vuori *et al*.^12^. Additionally, Zhang *et al*.^31^ have reported approximately 0.5 nm-thick PEG-SiCl coatings on silicon surfaces with smaller (600 Da) molecules. Because the length of an extended PEG 2000 would be closer to 10 nm, it is likely that the molecules were not standing in upright positions but instead spread out laterally on the surface^32^,^33^. Thus, they were able to effectively cover the entire surface even though the silane groups appeared to be loosely packed. The IEEB analysis is based on XPS data measured in ultra high vacuum conditions. The molecular brush type silane molecules may adopt a different surface orientation and apparent thickness in gaseous or liquid environment. Thus, the determined SPC overlayer thicknesses should be evaluated with caution. Still, densely packed overlayers would yield significantly higher thicknesses in the IEEB analysis. With denser packing, the silane-PEGs would have been forced to extend upwards which would have increased the thickness of the overlayer. This could possibly be achieved by using, for example, additional shorter spacer molecules to more effectively organize the silane-PEG molecules or by increasing the silane-PEG concentration^32^. Indeed, also here the higher SPC concentration of 5 mg/ml resulted in thicker SPC overlayer. However, in terms of chemical composition and surface coverage, no significant differences were observed between the two applied concentrations. Also, increasing the silane-PEG concentration further was found difficult due to solubility limits. Thus, the lower 3 mg/ml concentration was selected and used as the primary working concentration.

According to the contact angle measurements, silanization makes the SS surfaces more hydrophilic. For all the silane-modified samples, approximately 10-degree reductions in the contact angle values were observed compared to clean unmodified SS (see Supplementary Figure S2). This relatively small difference was found to be consistent between numerous samples and statistically significant (one-way ANOVA). In contrast, statistically significant differences between the two different silane-PEG-modified surfaces (SPC and SPB) were not found. Overall, the average contact angle for the unmodified SS reference was determined to be 48° ± 6°, whereas for SPC and SPB modified SS, the measured values were 39° ± 7° and 41° ± 3°, respectively. As more hydrophilic surfaces are often also considered to be more antifouling, the slightly higher hydrophilicity of the modified SS, thus, likely positively contributed to the improved biofouling resistance of the coated surfaces as was observed in the antifouling tests^34^,^35^. However, as the contact angle values of unmodified SS were also rather hydrophilic to begin with, it could not be unambiguously determined how significant the contribution of increased hydrophilicity was for the biofouling.

**Figure 3:**
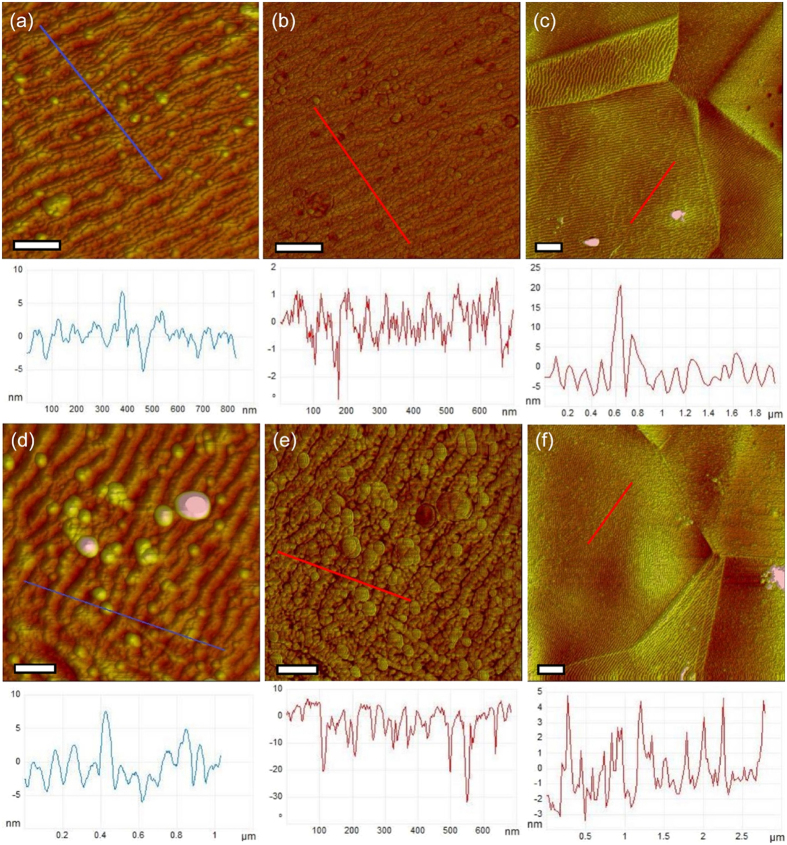
AFM images of SS-SPC. Typical AFM height (**a**) and phase (**b**) images of a silane-PEG-COOH-coated SS surface. Scale bars 200 nm. (**c**) Large scale image of the same sample as in (**a,b**). Scale bar 1 μm. (**d,e**) Height and phase images of an unmodified SS, respectively. Scale bars 200 nm. (**f**) A large-scale image of the same sample as in (**d,e**). Scale bar 1 μm.

In AFM, the silane-PEG overlayer could not be directly observed, apart from some occasional aggregates, but the typical topography and surface characteristics of SS were clearly visible in all the samples. This might be caused by the fact that, since the AFM imaging was performed in dry state, the conformation of the PEGs might have collapsed similarly to UHV conditions of the XPS measurements, which could make the coating extremely thin. Nonethless, the images concur with the PES analyses and imply that the overlayer is indeed very thin and mostly homogenous. However, in height images the SPC-modified surfaces appeared smoother than the unmodified SS, and phase images showed differences in the surface groove dips, which indicated that the dips were filled with material differing from SS. The representative images are shown in Fig. 3. Possibly, silane-PEG had accumulated in the grooves and partially filled them, which also agrees with the hypothesis of extensive lateral spreading of the surface-bound silane-PEGs as suggested by the IEEB analyses.

### Antifouling properties

**Figure 4:**
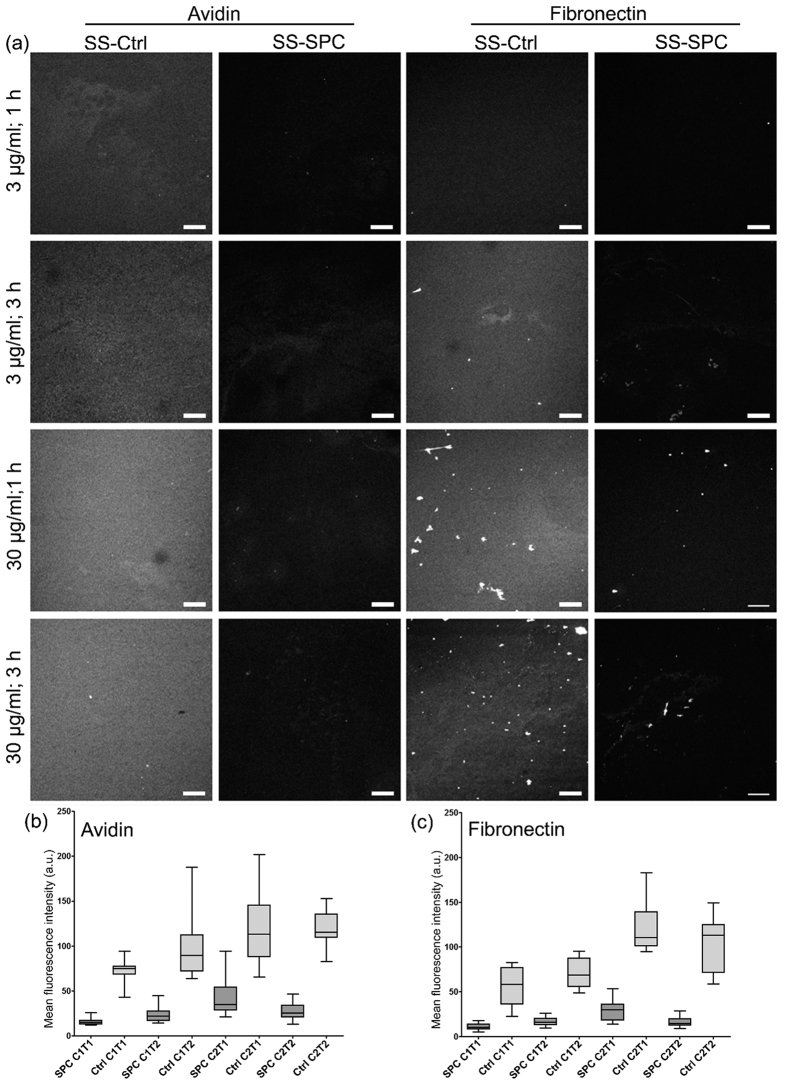
Protein adsorption on SS-SPC. (**a**) Fluorescence microscopy images of Alexa Fluor® 488 labeled avidin and fibronectin adsorbed on unmodified SS (SS-ctrl) and SS-SPC in different conditions. The used protein concentration was either 3 μg/ml (C1) or 30 μg/ml (C2), and the exposure time 1 h (T1) or 3 h (T2). Scale bars 100 μm. (**b,c**) The boxplots show the mean fluorescence intensities of the adsorbed avidin and fibronectin, respectively, on SS-SPC (SPC) and unmodified SS (Ctrl) in the conditions depicted in (**a**).

Surface-grafted PEG coatings are able to prevent biofouling of proteins and bacteria on distinct surfaces^31^,^36^,^37^,^38^. Therefore, the ability of the SPC-coated SS surfaces to resist biofouling was tested using two different types of protein, avidin and fibronectin, and *E. coli* bacteria. Two different incubation times and concentrations were used for both of the proteins, and in each case, the SPC overlayer significantly reduced biofouling on the SS surfaces (one-way ANOVA with Bonferroni post-hoc test, p < 0.05). For example, SS-SPC showed a 70% reduction in avidin (30 μg/ml) adsorption compared to the unmodified SS after an incubation time of 1 h. The respective reduction in the case of fibronectin (30 μg/ml) after 1 h of exposure was 72%. The corresponding fluorescence microscope images of the samples and the measured mean fluorescence intensities are shown in Fig. 4. However, the adsorption of the proteins could not be completely prevented, and the amount of adsorbed protein seemed to positively correlate with increasing incubation time (1 or 3 h) and protein concentration (3 or 30 μg/ml) to some extent. Nonetheless, even the highest detected average intensity values of the SS-SPC for both proteins stayed below the values observed from the unmodified SS under the mildest conditions. Altogether, the observed intensity values for both proteins were very similar, suggesting that even though avidin and fibronectin are very different in terms of structure and size, the coating was able to reasonably effectively reduce binding of both of the proteins. Although the recorded values for fibronectin appeared to be slightly lower, these could possibly be accounted by the lower fluorescence labeling densities per mass of protein that resulted from variations during sample preparation. Differences were detected in the arrangement of the proteins on the surface; avidin was typically evenly distributed, whereas fibronectin demonstrated aggregation and formation of fibril-like structures. Fibronectin clusters and fibrils were also more prominent in the samples with longer exposure times and higher protein concentrations. Spontaneous fibrillogenesis is characteristic for fibronectin, and it is known that fibronectin fibrils can be generated even in the absence of cells, for instance, utilizing a water-air interface^39^.

Similar reductions in protein adsorption have been reported, for example, by Yang *et al*.^38^ and Harder *et al*.^40^. Yang and colleagues coated SS with a poly(ethylene oxide)-poly(propylene oxide)-poly(ethylene oxide) (PEO-PPO-PEO) triblock copolymer, and presented that the coated SS surfaces were capable of significantly reducing the adsorption of bovine serum albumin (BSA). Moreover, they noticed that without initial conditioning of the SS with a surface hydrophobization step, comparable in terms of significance to the EC surface passivation method used here, the PEO-PPO-PEO simply adsorbed onto surfaces in ineffectual conformation and was unable to prevent non-specific protein adsorption. Harder and colleagues, in turn, created fibrinogen-resistant layers on gold surfaces utilizing oligo(ethylene glycol)-terminated self-assembled monolayers. Additionally, they observed that similar self-assembled monolayers on silver failed to resist fibrinogen adsorption as a consequence of differences in the conformation and arrangement of the monolayer assemblies. Therefore, the antifouling properties of PEG and PEG-like polymers appear to be conformation dependent. Hence, our positive results here provide indirect evidence for the proper attachment and arrangement of the SS-bound SPC.

**Figure 5:**
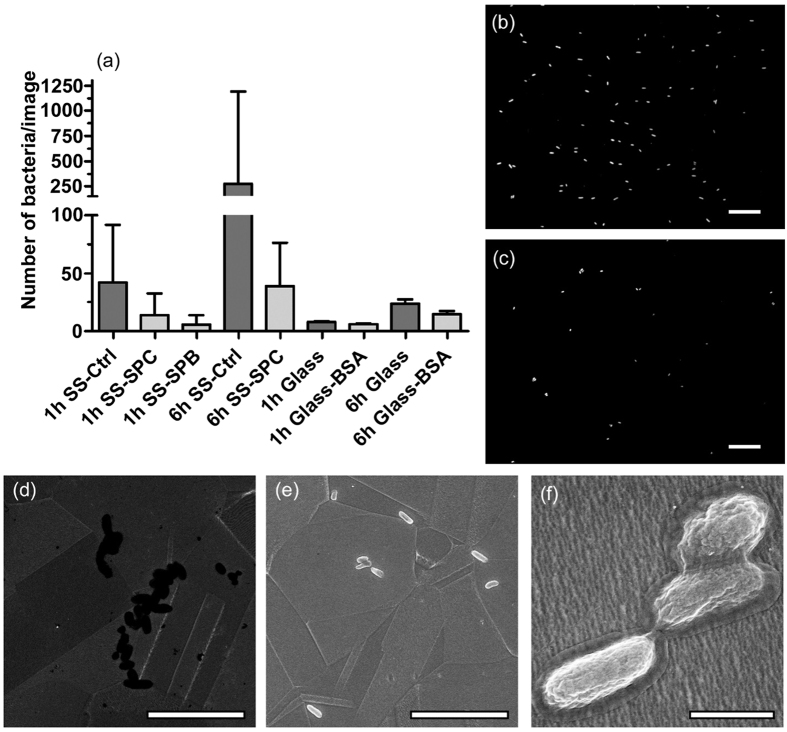
Attachment of *E. coli* on SS-SPC. (**a**) Amount (median with interquartile range) of tightly attached bacteria on unmodified SS (SS-Ctrl), SS-SPC and SS-SPB after 1 or 6 h exposure. Also values for unmodified and BSA-coated glass in similar conditions are shown for reference. (**b**) Representative fluorescence microscope image of *E. coli* on unmodified SS after 1 h of incubation. Scale bar 25 μm. (**c**) Representative fluorescence microscope image of *E. coli* on SS-SPC after 1 h of incubation. Scale bar 25 μm. (**d**) SEM image of *E. coli* on SS-SPC. The sample was not coated with conductive coating for imaging. Scale bar 10 μm. (**e**) SEM of bacteria on unmodified SS. The sample had been coated with AuPd prior to imaging. Scale bar 10 μm. (**f**) Higher magnification of the same sample as in (**e**). Scale bar 1 μm.

*E. coli* attachment was also significantly reduced on SS-SPC, and a statistically significant difference was found with both of the used time points (1 and 6 h) when compared to the unmodified SS (Kruskal-Wallis test with Dunn’s post-hoc test, p < 0.05). Unfortunately, complete prevention of bacterial attachment was not achieved. The bacteria were counted using fluorescence microscope images, and representative images along with SEM images are shown in Fig. 5. The median bacterial counts per image under different conditions were as follows: unmodified SS with 1 h exposure, 43 cells (Interquartile range (IQR) = 25.3–92.0 cells); SS-SPC with 1 h exposure, 14 cells (IQR = 3.6–32.4); unmodified SS with 6 h exposure, 892 cells (IQR = 512.0–1401.0); SS-SPC with 6 h exposure, 39 cells (IQR = 30.5–76.5). Thus, for 1 h exposure time, the SPC overlayer reduced bacterial adhesion by approximately 65%. Correspondingly, after exposure times of 6 h, the reduction in bacterial adhesion was observed to be close to 95%, which suggests the effect becomes more pronounced in longer time scales. Moreover, SS-SPB were also exposed to *E. coli* in the 1 h exposure test to examine if different functional end groups of silane-PEGs had an effect on the bacterial attachment. As a result, a comparable noticeable reduction in *E. coli* adhesion was observed on SS-SPB than on SS-SPC. The change was determined to be over 85%, which was in good accordance with the SS-SPC results. Thus, both the SPC and SPB overlayers demonstrated significant potential in preventing bacterial attachment and no notable effect due to the different end groups was detected.

SEM imaging also showed similar trend as unmodified SS had much higher amounts of attached *E. coli* than respective SS-SPC samples. On SS-SPC, the bacteria were typically found in a few clumps attached to each other, whereas on unmodified SS the bacteria tended to spread out more evenly, as is shown in Fig. 5(d–f). The topographical features of SS or defects in the SPC coating were expected to affect and promote bacterial attachment on the modified surfaces. However, apart from occasional alignment along the SS grain boundaries, no other preferential topographically-driven attachment was observed. Thus, direct conclusions cannot be drawn based on this data and further study is needed to elucidate the details of bacterial binding on modified SS.

Bacterial adhesion tests were also conducted on glass and BSA-coated glass to allow for the relative comparison of the silane-PEG-modified SS to a more well-known and generally utilized material. BSA-coated glass was used as a model for adsorption-resistive surface because BSA is routinely used as a blocking agent to prevent non-specific surface binding^41^. The results are shown in Fig. 5(a). Clean glass was observed to have approximately 80% less bacteria attached after 1 h of bacterial exposure than unmodified SS, and with the longer 6 h exposure the difference was even more pronounced. Thus, it seems that SS is markedly more susceptible to bacterial adhesion, which emphasizes the need for effective SS treatment. From the practical point of view, however, SS and glass are rarely readily interchangeable and are for totally different purposes, even though they are often used together to complement each other. As SS and glass are widely used materials in biotechnological laboratories, the test gives a good point of reference for the usability and potential of the modified SS in those settings.

### Biofunctionalization

**Figure 6:**
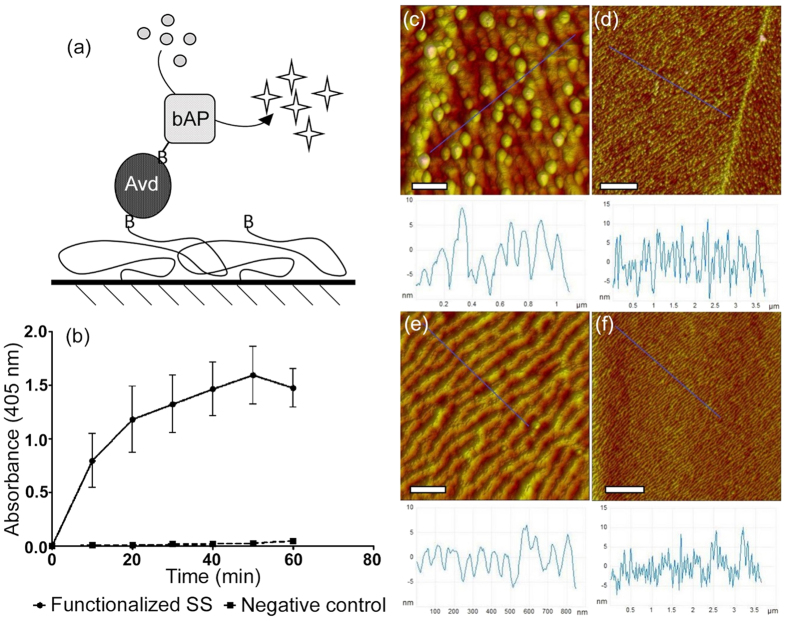
Biofunctionalization of SS-SPB. (**a**) Schematic illustration of the immobilization of bAP on SPB-modified SS via avidin-biotin bridge. (**b**) Spectrophotometric detection of the activity of surface-immobilized bAP. In the negative control, avidin binding sites had been blocked with free biotin to prevent selective attachment. (**c,d**) AFM images showing avidin functionalized SS-SPB. Scale bars 200 nm and 1 μm, respectively. (**e,f**) SS-SPB surface without avidin functionalization. Scale bars as in (**c,d**).

To examine the biofunctionalization potential of the silane-PEG-modified SS, SS-SPB substrates were manufactured, and neutral chimeric avidin was attached to the biotin groups of the coating. Biotin-avidin links are highly specific and their extremely high affinity (dissociation constant (K_d_) ~ fM) provides a route for a broad range of applications via attachment of biotinylated molecules^42^. Biotinylated alkaline phosphatase enzyme was linked to surface-bound avidin, as depicted in Fig. 6(a), and the functionalization effectivity was assessed by spectrophotometrically measuring the enzymatic activity of the immobilized bAP. In the negative control samples, avidin attachment was blocked with free biotin. The analysis revealed clear differences between the treatments, indicating successful selective attachment of bAP via avidin-biotin bonds, and simultaneous notable resistance against non-specific adsorption. The bAP enzymatic activity of the functionalized samples proceeded smoothly until approximately the 50 min time point, as shown in Fig. 6(b), where a plateau was reached. The absorbance at that time point was measured to be 1.59. On the other hand, the absorbance of the biotin-blocked controls remained close to zero (maximum average at the 60 min was 0.05). Thus, the SPB-coating was able to resist biofouling, while simultaneously allowing selective surface functionalization.

The attachment of avidin on SPB surfaces was also studied using AFM. Particles with a diameter of 5–10 nm were detected on the avidin treated SS-SPB, while they were absent from the negative control surfaces, as is shown in Fig. 6(c–f). As the size of an avidin molecule is close to 5 nm, the particles were most likely individual avidin, suggesting successful functionalization. The SS-SPB surfaces that had not been treated with avidin appeared similar to the SS-SPC surfaces shown in Fig. 3.

The data are in good accordance with the results reported previously in Vuori *et al*.^12^, where avidin-biotin technology was used to functionalize bimolecular organosilane-modified SS. Similarly, they used an AP enzymatic reaction to inspect the extent of functionalization and recorded absorbance values of 0.2 at 405 nm, which suggested successful functionalization. This value is significantly lower than the maximum values obtained in this study. However, values are not directly comparable with each other due to differences in the functionalization approaches used. While in Vuori *et al*. sparse and tunable functionalization of SS was aimed, a more straightforward and practical approach was pursued in this study. Nonetheless, it was stated that the surface functionalization was highly specific and mediated by avidin-biotin linkages because the presence of free biotin was shown to significantly inhibit the process. Similar strict specificity was also observed here.

Therefore, avidin-mediated functionalization can indeed be successfully applied to adjust the biocompatibility of silane-modified SS as long as free biotin groups are available on the surface. Here, significant functionalization efficiency was achieved by using SPBs. Furthermore, by optimizing the surface modification protocol and the presentation of the available surface-bound biotins, for example, by adjusting the length of the PEG chains or by mixing different polymers, even better results could be obtained. In the future, silane-PEG derivatives could effectively be used to immobilize surfaces with numerous other molecules and enzymes as well, as long as suitable linker pairs for functionalization are available. Hence, straightforward addition of a diverse range of abilities to SS could be achieved.

## Conclusions

Here, a straightforward solution-based method was used to deposit a silane-PEG overlayer onto SS. By using simple EC pretreatment followed by silanization, homogenous silane-PEG overlayers less than 10 Å in thickness with uniform and replicable chemical compositions were formed. The physicochemical properties of the overlayers were studied using XPS, AFM and contact angle measurements, whereas specific adhesion and enzyme activity tests were used to assess changes in biofunctionality. The silane-PEGs were found to efficiently cover the surfaces by laterally spreading across the samples. Thus, sparse and amorphous packing of the PEG chains was typically observed, and the protocol itself was found to be reliable and reproducible. In addition, the overlayers made SS slightly more hydrophilic and expressed significant antifouling potential by reducing the non-specific binding of avidin and fibronectin proteins by approximately 70% (measured for COOH-terminated silanes), and the attachment of *E. coli* by more than 65% (measured for both COOH- and biotin-terminated silanes). With further optimization of the protocol, the biofouling resistance could still most likely be improved. For example, mixing different types of polymers or PEGs of different sizes should affect the packing of the coating and alter the related antifouling properties.

By modifying SS with SPB, the addition of another layer of functionalization via avidin-biotin bridges was successfully performed without compromising the antifouling properties. Avidin was linked to the SPBs, and bAP was added thereupon to provide biofunctionality. By using avidin-biotin linkages, other biotinylated molecules could also be linked to SS-SPB, thus, providing an easily adjustable and generic method for fabricating applications such as customized biosensors, cell culture substrates or orthopedic implants bearing specific growth inducers or hormones. Furthermore, as long as suitable silane-PEG derivatives and molecules with respective linkers are available, other functionalization routes are possible. Therefore, the concept is not limited only to avidin-biotin technology, and it provides a solid and tailorable foundation for the extensive functionalization of SS.

## Additional Information

**How to cite this article**: Hynninen, V. *et al*. Improved antifouling properties and selective biofunctionalization of stainless steel by employing heterobifunctional silane-polyethylene glycol overlayers and avidin-biotin technology. *Sci. Rep.* **6**, 29324; doi: 10.1038/srep29324 (2016).

## Supplementary Material

Supplementary Information

## Figures and Tables

**Figure 1:**
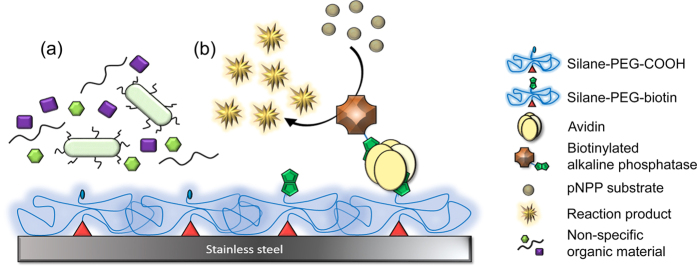
Schematic illustration of silane-PEG-modified SS. (**a**) SPC overlayer resists non-specific biofouling of both bacteria and proteins. (**b**) SPB overlayer enables selective functionalization of SS via avidin-biotin bridges with bAP without compromising the antifouling properties.
