# Supplementary material for: Improved antifouling properties and selective biofunctionalization of stainless steel by employing heterobifunctional silane-polyethylene glycol overlayers and avidin-biotin technology
Source: Sci Rep. 2016 Jul 6;6:29324. doi: 10.1038/srep29324 (PMC4933944; doi:10.1038/srep29324)
Supplement: Supplementary Information [file srep29324-s1.pdf]

Supplementary information

Improved antifouling properties and selective biofunctionalization of stainless steel by employing heterobifunctional silane-polyethylene glycol overlayers and avidin-biotin technology

Ville Hynninen[†], *Leena Vuori*[‡], *Markku Hannula*[‡], *Kosti Tapio*[§], *Kimmo Lahtonen*[‡], *Tommi Isoniemi*[§], *Elina Lehtonen*[‡], *Mika Hirsimäki*[‡], *J. Jussi Toppari*[§], *Mika Valden*[‡], *Vesa P. Hytönen*[†,⊥,*]

*corresponding author

[†]BioMediTech, University of Tampere, Biokatu 6, FI-33520 Tampere, Finland

[‡]Surface Science Laboratory, Optoelectronics Research Centre, Tampere University of Technology, PO Box 692, FI-33101 Tampere, Finland

[§]University of Jyvaskyla, Department of Physics, NanoScience Center, P.O. Box 35, FI-40014, University of Jyväskylä, Finland

[⊥]Fimlab Laboratories, Biokatu 4, FI-33520 Tampere, Finland

SURFACE COMPOSITION OF SS-EC AND SS-SPC SAMPLES DETERMINED BY XPS

Table S1 exhibits the relative elemental surface concentrations of the ethanol sonicated SS and SS-EC samples determined from the conventional XPS (hν = 1486.6 eV). The impurities on the ethanol sonicated SS samples are residues from the electrochemical polishing and atmospheric contamination. The EC treatment removes impurities from the surface, the amount of C and Si is decreased and all P is removed. The treatment leads to increased amount of sulphur, originates from the sulphate residue from the electrolyte solution.

Table S1. The relative elemental surface concentrations of SS and SS-EC samples as determined by XPS (hν= 1486.6 eV).

sample	C 1s (at.%)	O 1s (at.%)	Fe 2p (at.%)	Cr 2p (at.%)	Mn 2p (at.%)	Ni 2p (at.%)	Mo 3d (at.%)	N 1s (at.%)	Si 2p (at.%)	S 2p (at.%)	P 2p (at.%)
**SS**	19.4	47.6	8.0	12.7	3.5	1.7	0.9	1.6	2.5	0.5	1.6
**SS-EC**	14.1	48.1	13.0	12.1	4.0	2.1	1.5	1.9	0.3	2.8	-

Table S2 shows the relative elemental and chemical surface concentration of only the carbon, oxygen and silicon, i.e. the elements of the silane molecules, on SS-EC and SS-SPC samples. The elements that are only present in the SS-EC (Fe, Cr, Mn, Ni Mo and S) are omitted. According to the XPS analysis, no significant differences are observed on the relative elemental concentrations between the different SPC concentrations. Heating at 100 °C after the silanization may induce desorption of weakly bonded SPC molecules as the relative amount of Si is smaller on the samples examined with XPS after the heating.

Table S2. The relative elemental and chemical surface concentrations of C, O and Si SS-EC and SS-SPC samples as determined by XPS (hν= 1486.6 eV). The elements that are only present in the SS-EC (Fe, Cr, Mn, Ni, Mo, S) are omitted.

	C 1s (at. %)				O 1s (at. %)					Si 2p (at. %)
sample	C-C	C-O	C=O	C_{tot}	C=O	SO_4	OH	M-O	O_{tot}	
SS-EC	13.6	3.1	0.9	**17.7**	3.4	14.6	34.3	30.1	**82.4**	**0.0**
	C-C	C-O	C=O			C-O	OH	M-O		
SS-SPC 3 mg/ml	8.1	12.7	6.5	**27.2**		28.7	30.0	11.6	**70.3**	**2.6**
SS-SPC 3 mg/ml after heating	8.9	10.2	8.0	**27.0**		35.0	29.1	7.2	**71.3**	**1.6**
SS-SPC 5 mg/ml	9.5	11.8	5.5	**26.7**		25.1	35.3	9.6	**70.0**	**3.2**
SS-SPC 5 mg/ml after heating	9.5	8.9	8.4	**26.7**		23.6	38.1	9.7	**71.3**	**1.9**

SPC LAYER MORPHOLOGY DETERMINED BY INELASTIC ELECTRON ENERGY-LOSS BACKGROUND ANALYSIS

Determination of surface morphology, i.e., the depth distribution of elements and chemical compounds, from the XPS data was based on the inelastic electron energy-loss background (IEEB) analysis method developed by Tougaard.[1,2] It relies on description of photoelectron energy loss due to inelastic scattering, and thus allows non-destructive determination of surface morphology. The calculation is based on information on the thickness and coverage of the attenuating overlayer(s), initial energy of emitted electrons, inelastic electron mean free paths determined by the TPP-2M equation,[3] ionization cross-sections, measurement geometry and energy-dependence of the spectrometer transmission function.[1,2]

In our analysis, the 'Generate' program from QUASES software package[4] was utilized in conjunction with the 'islands: active substrate' concentration profile model. The analysis was simplified by restricting the number of islands to two: one for uniform thin layer, and one for

thicker clusters. In 'active substrate' mode the modelled signal originates from the substrate (Fe 2p of SS-EC) and is attenuated by the overlayer (SPC). The equations describing the attenuation in island structure morphology class are presented by Tougaard in Refs. (1) and (2). The maximum SPC layer thickness obtainable from the Fe 2p spectral region is approximately 20 nm, because the analysis depth of this method does not exceed 10λ.[1,2] Typical error estimate for the quantification by IEEB analysis is ± 15%.1 It should also be noted that the analysis technique does not specify whether the islands and clusters are protruding from the surface or if they are penetrating towards the bulk of the substrate, e.g., in case substrate is not flat and SPC fills up the depressions on the surface.

The method yields a modelled XPS spectrum generated from the measured SS-EC substrate signal (without overlayer) taking into account the attenuation to the signal by the SPC overlayer. The modelled spectrum contains the intensity and shape change to both the inelastic electron energy-loss background and photopeak of the original unattenuated SS-EC signal induced by the overlayer thickness, coverage and composition.

By varying the surface morphology of the model surface and by comparing the modelled spectrum to the experimental one, quantitative analysis of the SPC layer thickness was obtained. The correct morphology is found when the modelled spectrum overlaps throughout the spectrum's kinetic energy region with the one measured from a SS-SPC sample. The results are shown in Table S3.

Table S3. The surface morphology of SS-SPC samples as determined by IEEB analysis. The table shows the thickness and coverage of the thin SPC overlayer. The rest of the surface is covered with thick (>200 Å) clusters.

	thickness (Å)	coverage (%)
SS-SPC 3 mg/ml	4.9	88.2
SS-SPC 3 mg/ml after heating	4.6	86.9
SS-SPC 5 mg/ml	6.6	86.7
SS-SPC 5 mg/ml after heating	7.7	83.2

Figure S1 illustrates the IEEB analysis process for the sample SS-SPC 5 mg/ml. Fig. S1(a) contains the original measured XPS Fe 2p spectra of SS-EC and SS-SPC 5 mg/ml. Fig. S1(b) shows that uniform SPC layer morphology with any thickness does not yield a valid fit. Instead, a thin uniform SPC layer with thickness of 6.6 Å and coverage of 86.7% accompanied by thick clusters fits the data well as shown in Fig S1(c).

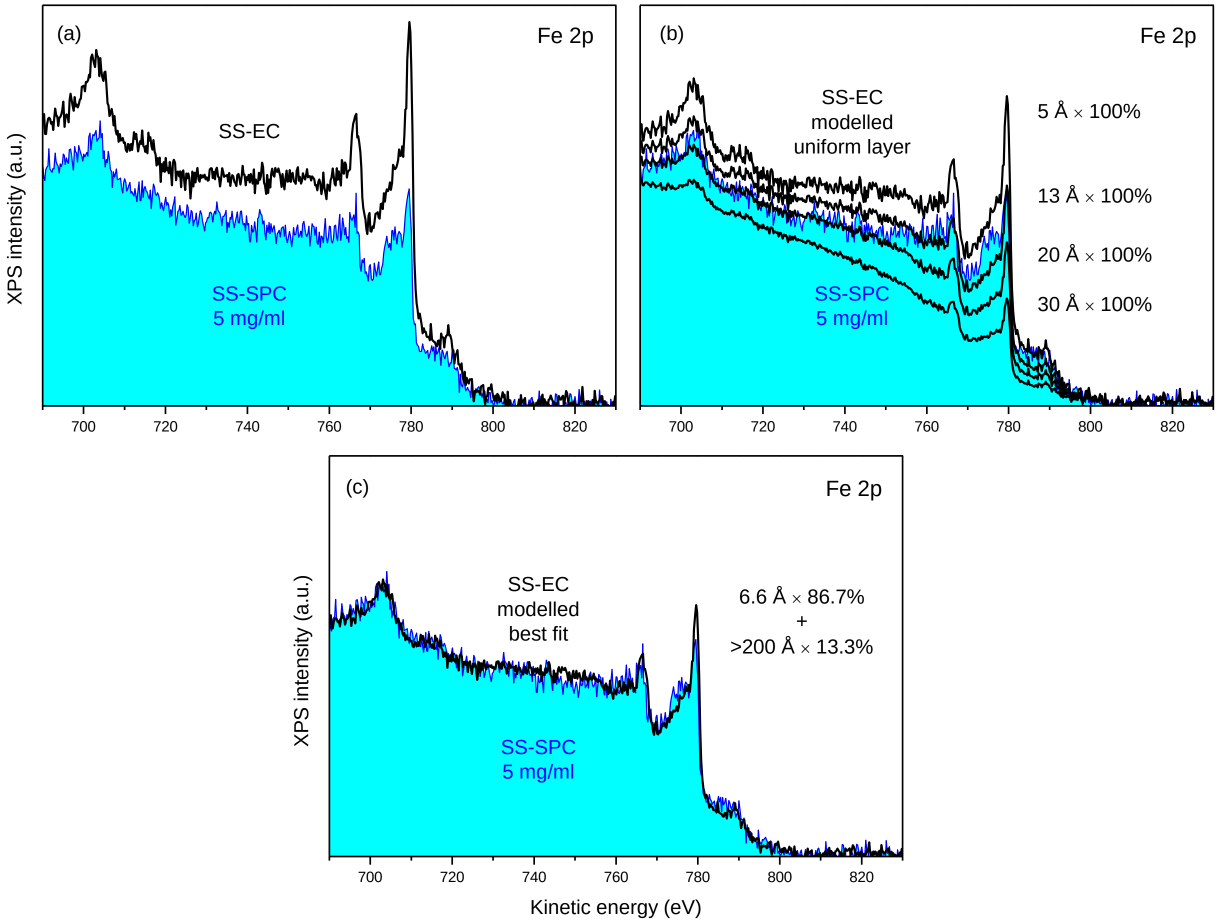

Figure S1. Inelastic electron energy-loss background (IEEB) analysis. The spectra were acquired with photon energy of 1486.6 eV.

(a) XPS Fe 2p spectra of SS-EC (black) and SS-SPC 5 mg/ml (blue). (b) Fittings using modelled uniform layer morfology with varying thickness. 5 Å: too thin layer (not enough attenuation). 13 Å: the main Fe 2p peak (780 eV) of the model spectrum (black) overlaps with the one measured from the SS-SPC sample (blue), but the background intensity (690–750 eV) is too high in the model spectrum. 20 Å: The backgrounds overlap, but the main Fe 2p peak intensity is too low in the model spectrum. 30 Å: Too thick layer (too much attenuation). Conclusion is that the SPC layer is not uniform. (d) Modelled best fit using non-uniform layer morphology.

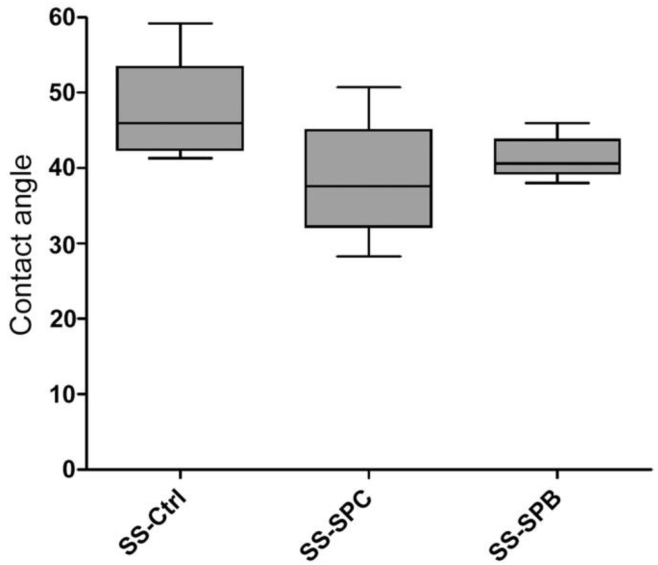

Figure S2. Contact angle values of clean stainless steel control (SS-Ctrl), silane-PEG-COOH modified stainless steel (SS-SPC) and silane-PEG-biotin modified stainless steel (SS-SPB).

REFERENCES

(1) Tougaard, S. Quantification of Nanostructures by Electron Spectroscopy. In *Surface analysis by Auger and X-ray photoelectron spectroscopy*; Briggs, D., Grant, J. T., Eds.; IM Publications and SurfaceSpectra Limited: United Kingdom, 2003; pp 295–345.

(2) Tougaard, S. Energy loss in XPS: Fundamental processes and applications for quantification, non-destructive depth profiling and 3D imaging. *J. Electron Spectrosc. Relat. Phenom.* **2010**, *178–179*, 128–153.

(3) Tanuma, S.; Powell, C. J.; Penn, D. R. Calculation of Electron Inelastic Mean Free Paths (IMFPs) VII. Reliability of the TPP-2M IMFP Predictive Equation. *Surf. Interface Anal.* **2003**, *35*, 268–275.

(4) Tougaard, S. QUASES: Software for Quantitative XPS/AES of Surface Nano-Structures by Analysis of the Peak Shape and Background – version 5.0; QUASES-Tougaard Inc.: Odense, Denmark, 2003. http://www.quases.com/.
